# Adeno-Associated Virus Vector Mediated Expression of an Oncogenic Retroviral Envelope Protein Induces Lung Adenocarcinomas in Immunocompetent Mice

**DOI:** 10.1371/journal.pone.0051400

**Published:** 2012-12-10

**Authors:** Nicolle M. Linnerth-Petrik, Lisa A. Santry, Darrick L. Yu, Sarah K. Wootton

**Affiliations:** Department of Pathobiology, Ontario Veterinary College, University of Guelph, Guelph, Ontario, Canada; University of Missouri, United States of America

## Abstract

Lung cancer is the most common cause of cancer-related death worldwide. A poor overall survival rate of 16% necessitates the need for novel treatment strategies. Mouse models of lung cancer are important tools for analyzing the significance of somatic mutations in the initiation and progression of lung cancer. Of additional importance, however, are animal models of virally induced cancers. JSRV is a simple betaretrovirus that causes contagious lung cancer in sheep known as ovine pulmonary adenocarcinoma and closely resembles human lung adenocarcinoma. Previously we showed that expression of the JSRV envelope (Env) from an AAV vector induced lung tumors in immunodeficient mice, but not in immunocompetent mice. Because of the importance of studying lung cancer in the context of an intact immune system we sought to improve our mouse model. In this report, we employed the use of a strong JSRV enhancer-promoter combination to express Env at high levels and demonstrate for the first time, lung tumor induction in immunocompetent mice. This occurred despite a robust Env-specific antibody-mediated immune response. The PI3K/Akt and MAPK pathways were activated in both immunocompetent and immunodeficient mice, however, differential activation of PTEN, GSKα, p70S6K, p38MAPK, ATF2 and STAT5 was observed. A JSRV Env lung tumor-derived cell line was shown to have a similar signal transduction activation profile as Env-induced lung tumors in C57BL/6 mice. Given the similarities between our model and pulmonary adenocarcinomas in humans, and the ease with which tumors can be induced in any transgenic mouse, this system can be used to uncover novel mechanisms involved lung tumorigenesis.

## Introduction

Lung cancer accounts for 28% of all cancer-related deaths each year making it the most common cause of cancer-related death worldwide (American Cancer Society, Cancer Facts and Figures 2012). Despite therapeutic advances, the overall 5-year survival rate for lung cancer is only 16%, indicating that novel treatment strategies are needed. An understanding of the genetic alterations involved in the initiation and progression of lung cancer would facilitate clinical treatment and early diagnosis. Indeed, the development of animal models harboring these genetic mutations has yielded valuable insights into the underlying molecular mechanisms of lung tumorigenesis and has provided important preclinical models for evaluating new drug therapies. Of additional importance, however, are animal models of virally induced cancers. The study of oncogenic retroviruses has provided much of the foundation for our current understanding of the genetic and molecular basis of cancer and these viruses continue to reveal important insights directly relevant to human cancer [1].

Jaagsiekte sheep retrovirus (JSRV) is a simple betaretrovirus that induces ovine pulmonary adenocarcinoma (OPA) in sheep [2]. OPA originates in alveolar type II cells of the peripheral lung [3] and displays papillary, acinar and bronchioloalveolar features similar to human pulmonary adenocarcinoma, particularly that of non-smokers [4], [5]. Unlike most replication-competent retroviruses that cause cancer by insertional activation of cellular proto-oncogenes or through acquisition of cellular proto-oncogenes, the envelope (Env) glycoprotein of JSRV is itself oncogenic and represents a newly evolved mechanism of transformation [6], [7]. While the phosphatidylinositol 3-kinase (PI3K)/Akt [8]–[11] and mitogen-activated protein kinase (MAPK) [12]–[14] pathways have been implicated in the transformation by JSRV Env, very little is known about the exact mechanism by which Env engages these signal transduction pathways to initiate transformation, nor have these pathways been evaluated extensively *in vivo*.

We previously showed that intranasal delivery of a replication-defective adeno-associated virus (AAV) vector expressing the JSRV Env protein from a Rous sarcoma virus (RSV) promoter induced lethal lung tumors in immunodeficient mice in as little as 5 months post-infection [6]. Despite JSRV Env expression in all lung epithelial cells, tumors were derived exclusively from alveolar type II cells and displayed histological similarities to human lung adenocarcinomas. While lethal disease was observed in 100% of immunodeficent mice that received the vector, immunocompetent mice mounted a strong antibody-mediated immune response against JSRV Env and did not develop tumors. Here we test the hypothesis that C57BL/6 mice infected with A_JE_JJenv, an AAV vector expressing JSRV Env from its cognate LTR promoter in conjunction with a newly identified JSRV-derived enhancer [15], will develop lung tumors with a similar signal transduction profile to human lung adenocarcinoma, particularly that of never smokers [16]. We show that immunocompetent mice infected with this enhanced Env expressing AAV vector develop lung tumors by 2.5 months post-infection and that the Akt, Erk1/2, EGFR and STAT3 pathways are activated in these tumors. Additionally, we have characterized the growth and signal transduction properties of the JSRV Env-induced lung tumor cell line, RJenvC1, revealing a similar pattern to that observed *in vivo*, making this a useful *in vitro* tool with which to dissect the mechanisms of Env-induced lung tumorigenesis. By understanding how JSRV Env induces lung cancer we stand to uncover new and possibly unexplored mechanisms involved in the initiation and progression of lung cancer in humans.

## Materials and Methods

### Ethics Statement

All mouse experiments were performed in compliance with the guidelines set forth by the Canadian Council on Animal Care (CCAC). The protocol was approved by the Animal Care Committee of the University of Guelph (Animal Utilization Protocol: 09R072). All vector administrations were performed under isoflurane anesthesia, and all efforts were made to minimize suffering.

### Cell Culture

HEK 293 cells (ATCC CRL-1573) were propagated in Dulbecco's modified Eagle medium supplemented with 10% fetal bovine serum, 2 mM L-glutamine and 1% penicillin/streptomycin. Cells were maintained at 37°C in 5% CO_2_. The Rag2/normal lung epithelial (RNLE) cell line was generated using techniques described previously [17]. RJenvC1 (a kind gift from Dr. Dusty Miller, Fred Hutchinson Cancer Research Center) [17] and RNLE cells were maintained on FNC (AthenaES) coated plates grown in a 1∶1 ratio of 3T3 conditioned medium:Keratinocyte-SFM (KSFM, Gibco BRL).

### AAV Vectors

**Figure 1:**
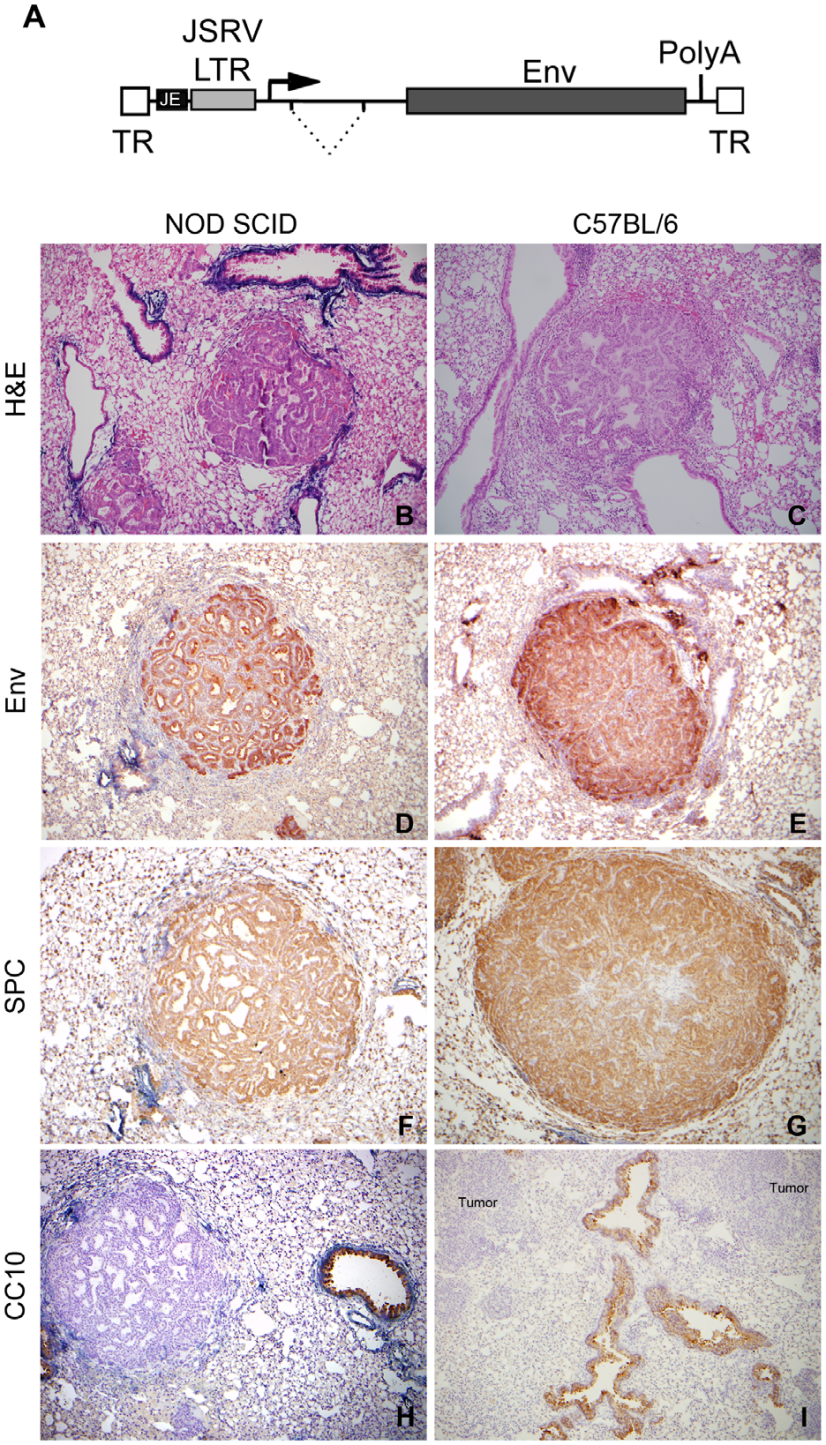
AAV mediated expression of JSRV Env induces lung adenocarcinomas in immunocompetent mice. Illustration of A_JE_JJenv, the AAV vector used to express JSRV Env in mouse lungs (A). TR, AAV terminal repeat; JE, JSRV enhancer element; Env, JSRV envelope; and PolyA, polyadenylation signal. Immunostaining for Env, SPC, and CC10 expression in lung tissue from A_JE_JJenv infected NOD/SCID (B, D, F, H) and C57BL/6 (C, E, G, I) mice at 10x magnification. Representative images of hematoxylin and eosin stained lung tumors from NOD/SCID (B) and C57BL/6 (C) mice. Representative images show robust Env expression within all cells of the lung tumor (D, E). Representative images show lung tumors staining uniformly positive for SPC, a marker for alveolar type II cells (F, G), and negative for CC10, a marker for Clara cells (H, I; unstained tumors are labeled).

A recombinant AAV vector containing an expression cassette for JSRV Env and serotype 2 inverted terminal repeats (ITRs) was generated by standard cloning techniques (A_JE_JJenv, Figure 1A). Briefly, the JSRV LTR-enhancer was amplified from the molecular clone of JSRV (pCMVJS_21_ [18], kindly provided by Dr. Massimo Palmarini, University of Glasgow) using the following forward 5'-CGTCTAGAGCTGCATATGAAATATAGAAATAGGTTACAGCACC-3' and reverse 5'-ATAAAGCTTCCTGCCGCGGCCAGCACAAG-3' primers. To generate A_JE_JJenv, the JSRV LTR-enhancer was digested with *Xba*I and *Hind*III and cloned into ARJenv [6] to replace the RSV LTR. The packaging/helper plasmid, pDGM6 [19], which encodes the serotype 6 capsid, was kindly provided by Dr. David Russell (University of Washington). AAV vectors and packaging plasmids were propagated in *Escherichia coli* GT116 (InvivoGen). AAV vectors were produced by cotransfection of HEK 293 cells with vector and packaging plasmid as described previously [20]. AAV vector titers were determined by Southern blot [21].

#### **AAV vector delivery to mouse airways**

Seven-week-old C57BL/6 and NOD/SCID male mice (Charles River Laboratories) were lightly anesthetized and given 1 x 10^11^ vector genomes (vg) of A_JE_JJenv and 5 x 10^9^ vg of ARAP4 in 3 x 50 µl doses via intranasal administration. Mice were euthanized at 2.5 and 6 months post vector administration (5 mice per time point). Lung and organ tissues were either flash frozen in liquid nitrogen or fixed in 2% paraformaldehyde and paraffin embedded.

### ELISA for the Detection of Serum Antibodies against the JSRV Env Protein

Mouse serum was monitored for the development of antibodies against the JSRV Env protein using an antigen-specific ELISA as described previously [22].

### Immunohistochemistry

Sections of paraffin embedded tissue were subjected to immunohistochemical staining as described previously [23].

### Western Blot Analysis

Monoclonal antibodies specific for phospho-EGFR (Tyr^1068^; Tyr^1148^), Akt, phospho-Akt (Thr^308^), phospho-Akt (Ser^473^), phospho-PDK1 (Ser^241^), phospho-GSK-3α/β (Ser^21/9^), phospho-Cyclin D1 (Thr^286^), phospho-p44/42 MAPK (Thr^202^/Thr^204^), p44/42 MAPK, PTEN, phospho-PTEN (Ser^380^), phospho-mTOR (Ser^2448^), phospho-p70S6K (Thr^389^), phospho-p38 MAPK (Thr^180^/Tyr^182^), phospho-ATF-2 (Thr^71^), phospho-STAT3 (Ser^727^), phospho-STAT3 (Tyr^705^), phospho-STAT5 (Tyr^694^), phospho-c-Fos (Ser^32^) and Cox2 were purchased from Cell Signaling Technology. Antibodies against surfactant protein C (SPC), Clara cell 10 (CC10) protein and actin were purchased from Santa Cruz Biotechnology. Anti-Env monoclonal antibody was described previously [22]. Lung tissue was homogenized in RIPA buffer (50 mM Tris pH7.5, 150 mM NaCl, 1% Triton X-100, 0.1% SDS, 10 mM EDTA, 1% sodium deoxycholate) containing Na_3_VO_4_ (1 mmol/L), NaF (50 mM) and a cocktail of protease inhibitors (Sigma). Cell lysates were separated by gel electrophoresis (12% Tris- glycine gel) and transferred to PVDF membranes. Membranes were blocked in 5% skim milk-PBST and primary antibodies were incubated at a dilution of 1∶1000 in 1% BSA-PBST overnight at 4°C. Proteins were detected using HRP-conjugated secondary antibodies at a 1∶2000 dilution (Invitrogen) and Western Lightning Chemiluminescence substrate (Perkin-Elmer). Images were captured using x-ray film.

### Determination of Cell Viability and Growth in the Absence of Serum

RNLE or RJenvC1 cells (7×10^4^) were seeded onto six-well FNC (AthenaES) coated plates and grown to 60% confluence. Serum starvation was initiated by washing cells and replacing with medium lacking serum and growth factors. Cells were harvested at four time points (three wells per time point) and the number of viable cells as determined by Trypan blue exclusion were counted in triplicate.

### Anchorage-Independent Growth in Soft Agar

RJenvC1 and RNLE cells (5000 cells/well) were plated in a six-well dish in medium containing 0.35% Ultrapure LMP-agarose (Gibco) on an underlay of 0.5% agarose and cultured at 37°C with 5% CO_2_. The culture was analyzed in triplicate. Colonies were stained with crystal violet and imaged after four weeks.

### Immunofluorescence Microscopy

RNLE and RJenvC1 cells were plated onto FNC-coated coverslips. Cells were fixed with 3.7% paraformaldehyde-PBS for 10 minutes, permeablized with 0.2% Triton X-100 in PBS and blocked with 5% BSA-PBS. Coverslips were incubated overnight at 4°C with a 1∶50 dilution of antibodies specific for SPC and CC10. After washing, cells were incubated with Alexa Fluor 488-conjugated secondary antibody (1∶100; Invitrogen) for 1 hour and mounted using Prolong Gold with DAPI (Invitrogen). Images were captured using a Carl Zeiss Axio 154 Observer A1 inverted fluorescence microscope.

## Results

### Expression of JSRV Env from its Cognate LTR Promoter-enhancer Induces Lung Tumors in C57BL/6 Mice

An AAV vector expressing JSRV Env from the JSRV LTR-enhancer (A_JE_JJenv; Figure 1A) was constructed in order to optimize Env expression in the airway epithelium of mouse lungs. A mixture of 1×10^11^ vg of A_JE_JJenv and 5×10^9^ vg of an AAV6 vector that expressed human placental alkaline phosphatase (ARAP4 [24]) was administered to the noses of lightly anaesthetized 7-week-old C57BL/6 and NOD/SCID mice. NOD/SCID mice were included to control for oncogenicity of the A_JE_JJenv vector and the ARAP4 vector was included to control for transduction efficiency. After 2.5 months, tumor development could be observed in 100% of mice. H&E staining of lung sections at 6 months post-infection revealed the presence of multiple papillary alveolar/bronchiolar adenomas that grew along the alveolar walls and projected into the alveolar septa (NOD/SCID; Figure 1B, C57BL/6; Figure 1C) as well as a large number of well-circumscribed adenocarcinomas that in some instances compressed the surrounding parenchyma (Figure 1G). Interestingly, there did not appear to be any obvious differences in tumor number, size or histopathology between the two strains of mice. All lung tumors expressed Env (Figure 1D and E) and SPC (Figure 1F and G), a marker for alveolar type II cells. No tumors stained positive for CC10, a marker for bronchiolar non-ciliated Clara cells (Figure 1H and I). No metastasis or JSRV Env expression was observed in any of the other organs examined.

**Figure 2:**
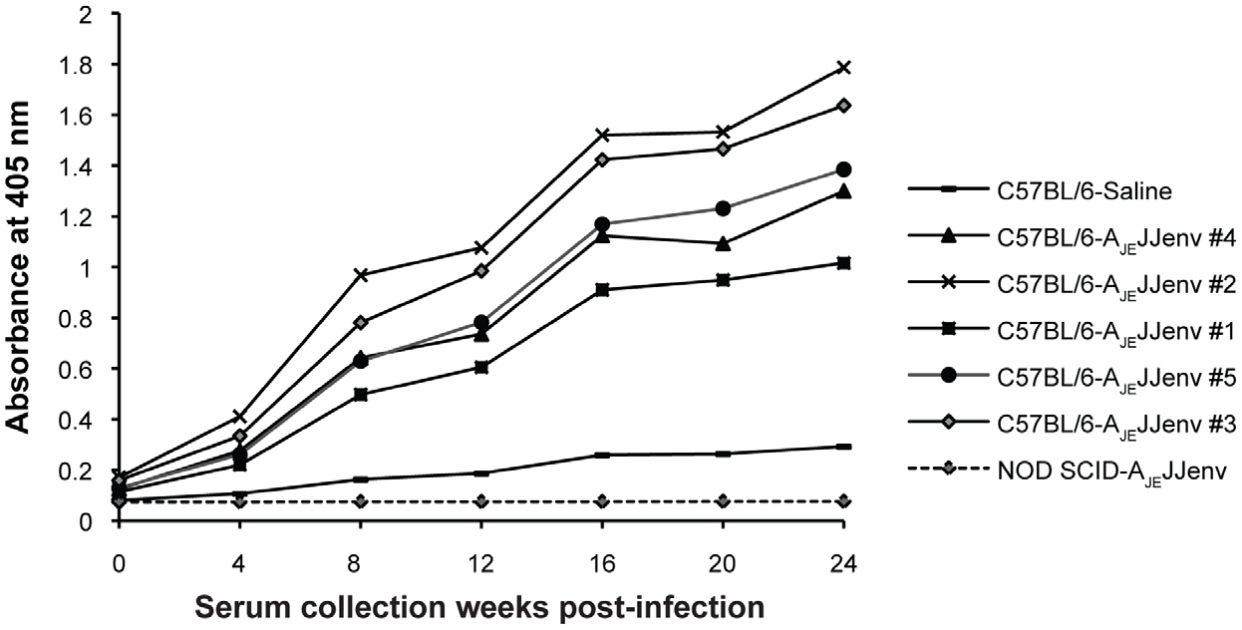
Antibody-specific immune response to JSRV Env in A_JE_JJenv infected C57BL/6 mice. A_JE_JJenv infection of C57BL/6 mice results in Env-specific antibody production that increases over time. Serum was collected every 4 weeks up to 24 weeks and subjected to antigen-specific ELISA. Total IgG was detected using a peroxidase-conjugated anti-mouse secondary antibody. Individual points represent the mean of triplicate wells.

To evaluate whether C57BL/6 mice infected with A_JE_JJenv mounted an immune response against JSRV Env, serum was collected every 4 weeks for a total of 24 weeks and tested in an antigen-specific ELISA. All C57BL/6 mice elicited an immune response against Env that increased over time (Figure 2). Interestingly, mice with the greatest tumor burden had the highest Env antibody titers (e.g. mouse #2). Taken together, these results indicate that expression of Env from its cognate LTR-enhancer can induce lung adenocarcinomas comprised of alveolar type II cells in immunocompetent mice and that despite a robust antibody-mediated immune response against Env, tumorigenesis cannot be controlled by the normal immune system of these mice.

### JSRV Env Activates the PI3K/Akt Signaling Pathway

**Figure 3:**
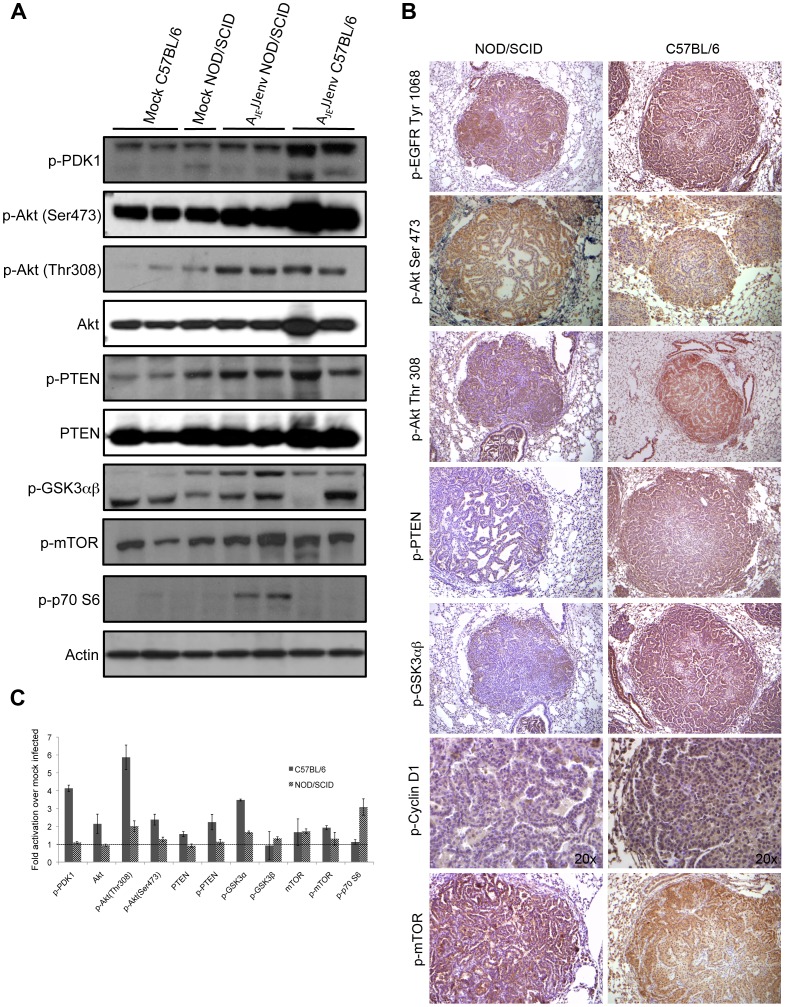
JSRV Env activates the PI3K/Akt pathway. (A) Representative western blot analysis of 80 µg protein extracts from the lungs of A_JE_JJenv mice at 20 weeks post-infection using a panel of phospho-specific (p) antibodies. (B) Representative immunohistochemical staining of lung sections from A_JE_JJenv infected mice at 20 weeks post-infection at 10x magnification unless otherwise stated. Note that the dark blue staining in some of the images represents alkaline phosphatase. (C) The ratio of protein over β-actin signal intensity was determined for each band on western blot using densitometric analysis and the average fold activation over mock infected was graphed. Values above the dotted line represent activation above mock infected.

Signaling pathways involved in JSRV Env-mediated lung tumor development in C57BL/6 and NOD/SCID mice were examined using a combination of western blot analysis and immunohistochemical staining. NOD/SCID mice were included in the analysis to determine whether JSRV Env-activated signal transduction pathways differed in the absence of a functioning immune system. Epidermal growth factor receptor (EGFR) is a member of the ErbB family of tyrosine kinase receptors that stimulates a number of different signal transduction pathways, including the PI3K/Akt pathway and the Ras/MAPK pathway [25]. Both the C57BL/6 and NOD/SCID lung tumors were uniformly positive for phosphorylated EGFR at Tyr1068 (Figure 3B), whereas there was a complete lack of phosphorylation at Tyr1148 (data not shown). Examination of the Akt pathway revealed that while Akt was activated via phosphorylation of Ser473 and Thr308 in both C57BL/6 and NOD/SCID lung tumors (Figure 3A and B), an increase in the phosphorylation of the upstream signaling molecule phosphoinositide-dependent kinase 1 (PDK-1) was only observed in immunocompetent C57BL/6 mice (Figure 3A). There were no differences in the protein levels of the tumor suppressor PTEN in tumor and control lungs (Figure 3A), but there appeared to be more activated PTEN in the C57BL/6 mouse lung tumors (Figure 3B). Immunohistochemical staining with a phospho-PTEN specific antibody showed strong localization to cells in the periphery of the tumors in the C57BL/6 mice, but very little staining in the NOD/SCID lung tumors (Figure 3B). Glycogen synthase kinase-3α/β (GSK3α/β), a critical downstream component of the PI3K/Akt cell survival pathway, showed slightly higher activation of both α and β subunits in NOD/SCID mice, but only the α subunit appeared to be activated in C57BL/6 lung tissue (Figure 3A). Interestingly, the C57BL/6 lung tumors showed uniform intense pGSK3α/β staining, while only the periphery of the NOD/SCID lung tumors stained positive for p-GSK3α/β (Figure 3B).

The cyclin D1 proto-oncogene is an important regulator of G1 to S-phase transition and is frequently overexpressed in NSCLC tumours and pre-invasive bronchial lesions [26]. As shown in Figure 3B, cyclin D1 was activated in both NOD/SCID and C57BL/6 Env-induced lung tumors, with the latter displaying much more intense staining.

The mammalian target of rapamycin (mTOR), a key player in cell growth and tumor development located downstream in the Akt signaling pathway, was activated in Env-induced lung tumors of both C57BL/6 and NOD/SCID mice (Figure 3A, B). Despite mTOR activation in both mouse strains, the serine/threonine kinase p70S6K, one of the key substrates of activated mTOR, was only activated in NOD/SCID mice (Figure 3A). Taken together, these results indicate that the JSRV Env protein is a potent activator of the PI3K/Akt pathway in both C57BL/6 and NOD/SCID mice. The most notable differences between the two strains of mice were the strong activation of PDK-1 and Akt (Thr308) in C57BL/6 mice and the lack of p70S6K activation in C57BL/6 lung tumors (Figure 3C).

### JSRV Env Activates MAPK Signaling Pathways

**Figure 4:**
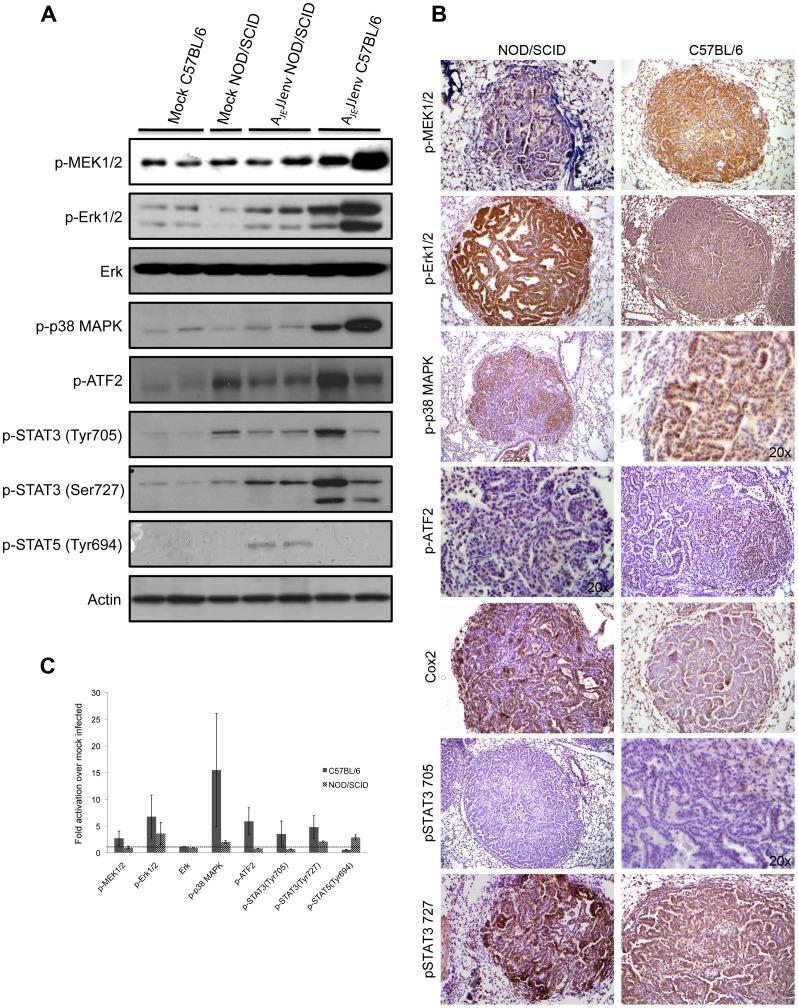
JSRV Env activates the MAPK pathway. (A) Representative western blot analysis of 80 µg protein extracts from the lungs of A_JE_JJenv mice at 20 weeks post-infection using a panel of phospho-specific (p) antibodies. (B) Representative immunohistochemical staining of lung sections from A_JE_JJenv infected mice at 20 weeks post-infection at 10x magnification unless otherwise stated. Note that the dark blue staining in some of the images represents alkaline phosphatase. (C) The ratio of protein over β-actin signal intensity was determined for each band on western blot using densitometric analysis and the average fold activation over mock infected was graphed. Values above the dotted line represent activation above mock infected.

MAPK pathways have been shown to play a key role in the transduction of extracellular signals that lead to proliferation and cell survival. In mammalian cells, three MAPK families have been identified: extracellular signal-regulated kinase 1 and 2 (ERK1/2), stress-activated protein kinase/c-Jun amino terminal kinase (SAPK/JNK), and p38 MAPK [27]. The Erk1/2 pathway was activated in both C57BL/6 and NOD/SCID Env-induced lung tumors, with higher levels of MEK1/2 and Erk1/2 activation in C57BL/6 tumors (Figure 4A and B). Additionally, the p38 MAPK pathway was highly activated in C57BL/6 lung tumors (Figure 4A). By immunohistochemical staining, p38 MAPK appeared to be activated in NOD/SCID lung tumors (Figure 4B), however this did not correlate well with western blot results, which suggested a modest increase in p38 MAPK activation (Figure 4A). The transcription factor ATF2 is a target of the p38 MAPK signaling pathway and was highly activated in C57BL/6 Env-induced lung tumors (Figure 4A), whereas there was no evidence of increased ATF2 phosphorylation in NOD/SCID lung tumor tissue (Figure 4A). Immunohistochemical staining showed nuclear phosph-ATF2 localization in patches of cells within both NOD/SCID and C57BL/6 Env-induced lung tumors (Figure 4B), with greater activation in the latter.

The STAT3 transcription factor is an important signaling molecule in a number of human cancers, including lung cancer [28]. Persistently activated STAT3 and to some extent STAT5, increase tumor cell proliferation, survival and invasion while suppressing anti-tumour immunity [29]. STAT3 was strongly phosphorylated at Tyr727 in both C57BL/6 and NOD/SCID lung tumor tissue (Figure 4A) and immunohistochemical staining was localized to a high percentage of cells within the tumors (Figure 4B). Conversely, only weak phosphorylation of STAT3 on Tyr705 was observed immunohistochemically (Figure 4B). Interestingly, activation of STAT5 was only measurable in tissues extracted from NOD/SCID lung tumor bearing mice (Figure 4A).

A growing body of evidence indicates that the anti-apoptotic protein cyclooxygenase 2 (COX-2) plays a key role in lung cancer, and may serve as a potential marker of prognosis in this disease [30]. COX-2 was overexpressed in both C57BL/6 and NOD/SCID Env-induced lung tumors, with more intense staining observed in NOD/SCID lung tumors (Figure 4B). Lastly, we investigated whether the oncogene c-fos, which promotes quiescent cells to enter the cell cycle [31], was activated in Env-induced lung tumors. Indeed, phosphorylated c-fos was detected exclusively in the nucleus of C57BL/6 lung tumors, however, there was a complete absence of staining in the NOD/SCID lung tumors (data not shown).

Taken together, these results demonstrate that both the ERK1/2 and p38 MAPK signal transduction pathways are involved in JSRV Env-mediated lung tumorigenesis in C57BL/6 mice and that activation of STAT5 is unique to NOD/SCID mice (Figure 4C).

### The PI3K/Akt and MAPK Pathways are Similarly Activated in a JSRV Env-induced Mouse Lung Tumor Cell Line

**Figure 5:**
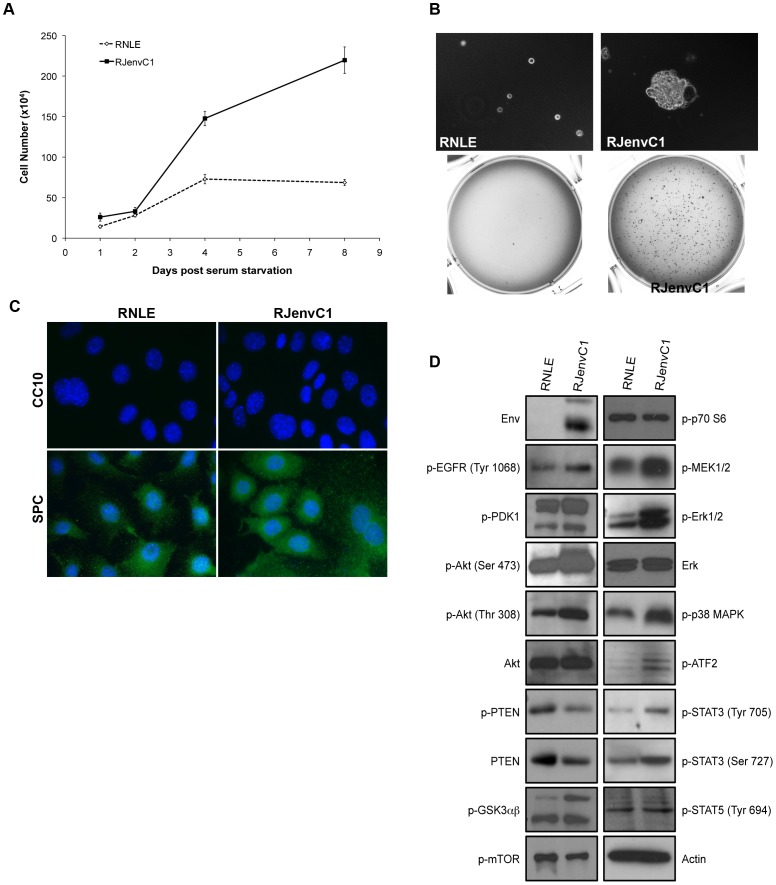
*In vitro* characterization of the JSRV Env-induced mouse lung tumor derived cell line, RJenvC1. (A) RJenvC1 cells grow in the absence of serum. RJenvC1 and RNLE cells were cultured for 24 h in complete medium containing serum. Culture medium was then removed and replaced with serum-free culture medium. At 24, 48, 96 and 192 hrs post-serum starvation, cells were assayed for viability by Trypan blue staining and total cell counts were determined. Graphed results represent the average of 3 independent assays. (B) RJenvC1 cells form colonies in soft agar whereas RNLE cells do not. Top panel, 20x magnification. (C) RJenvC1 and RNLE cells express markers of alveolar type II cells. RJenvC1 and RNLE cells were SPC positive and CC10 negative (Images are depicted at 100x magnification). Isotype–matched antibodies at equivalent concentrations did not reveal any immunofluorescence staining (data not shown) (D) The PI3K/Akt and MAPK pathways are activated in RJenvC1 cells in comparison to normal RNLE cells. Representative western blot analysis of 80 µg protein extracts from RJenvC1 and RNLE cells using a panel of phospho-specific (p) antibodies.

Orthotopic transplantation of RJenvC1 cells, a cell line derived from JSRV Env tumor bearing C57BL/6 Rag2 mice [17], efficiently induces lung tumors in immunodeficient mice and thus represents a valuable tool with which to dissect the contribution of individual signal transduction pathways. Therefore, we sought to compare RJenvC1 cells with that of JSRV Env-induced lung tumors to evaluate how well this tumor cell line parallels signaling *in vivo*. In addition, we generated a normal mouse lung epithelial cell line (RNLE) and characterized these cells in conjunction with RJenvC1 cells for their ability to grow in the absence of serum and form colonies in soft agar. As expected, the RJenvC1 cells displayed robust growth in the absence of serum or additional growth factors, whereas RNLE cell growth plateaued at 4 days post serum starvation and ceased to divide thereafter (Figure 5A). We next assessed the ability of RJenvC1 to form colonies in semisolid agarose medium. RJenvC1 colonies were visible by 9 days post-seeding but no RNLE colonies developed (Figure 5B). Thus, RJenvC1 cells display anchorage-independent growth, which, as demonstrated by the ability of RJenvC1 cells to form lung tumors in orthotopically transplanted mice, correlates with tumorigenic potential *in vivo*. As was observed for the JSRV Env-induced lung tumors, the RJenvC1 and RNLE cell lines express SPC but not CC10 (Figure 5C) indicating that these cells have retained their alveolar type II phenotype in culture.

Lastly, to evaluate whether the RJenvC1 cells maintain the same signal transduction pattern as JSRV Env-induced lung tumors, western blot analysis of key molecules in the PI3K/Akt and MAPK signaling pathways was conducted. JSRV Env was constitutively expressed only in the RJenvC1 cell line and similar to the C57BL/6 JSRV Env-induced lung tumors, the PI3K/Akt, Erk1/2 and p38 MAPK pathways were activated in RJenvC1 cells (Figure 5D). RJenvC1 cells showed higher levels of Akt phosphorylation at Ser473 and Thr308 relative to normal RNLE cells but expressed lower levels of PTEN in comparison to RNLE cells. Higher amounts of activated GSK3α were observed in the RJenvC1 cells, with little difference in GSK3β activation between these and RNLE cells. Similar to the *in vivo* model, elevated levels of activated MEK1/2, ATF-2 and STAT3 (Tyr705 and Tyr727) were observed in the RJenvC1 tumor cells, with no difference in STAT5, mTOR or p70S6K activation. Altogether, these data indicate that the RJenvC1 tumor cell line retains its transformed, alveolar type II phenotype in culture and has a similar signal transduction pathway activation signature as JSRV Env-induced lung tumors in C57BL/6 mice.

## Discussion

In the present study, we demonstrate that AAV vector-mediated expression of the JSRV Env protein from its cognate LTR-enhancer results in rapid and efficient lung tumorigenesis in C57BL/6 mice even in the presence of an antibody-mediated immune response against Env. Tumor formation was observed in 100% of mice as early as 2.5 months post-infection and resulted in lethal lung tumors between 5–6 months post-infection. Constitutive Env expression was confirmed in all mouse lung tumors. Despite the potential for JSRV Env expression in the entire respiratory epithelium [15], all lung tumors were SPC positive suggesting that the tumors originated from alveolar type II cells or a progenitor cell that expresses SPC. The PI3K/Akt, Erk1/2 and p38 MAPK signal transduction pathways were activated in both C57BL/6 and NOD/SCID mice infected with A_JE_Jenv; however, a higher overall level of activation was observed in the immunocompetent mice. The RJenvC1 lung tumor cell line was able to proliferate in serum free conditions, form colonies in soft agar and maintain signaling through the Akt, Erk1/2, and p38 MAPK pathways, making this an ideal *in vitro* model system to study the mechanisms of JSRV Env-mediated transformation.

Several rodent models have been established in an attempt to understand the biology of JSRV Env-mediated tumorigenesis [6], [32], [33]. The first mouse model to demonstrate the oncogenic potential of Env involved intranasal delivery of an AAV6 vector expressing JSRV Env from a RSV promoter [6]. This vector induced lung tumors in immunodeficient mice, but not immunocompetent mice. Next, a transgenic mouse model was created whereby an HA epitope-tagged version of JSRV Env was expressed under the control of the surfactant protein A (SPA) promoter [32]. This mouse model showed low Env expression in the lung and the only tumors observed to express the transgene were subdermal lipomas. In 2009, a transgenic mouse model expressing JSRV Env under the control of an SPC promoter in FVB/N mice was generated [33]. At 6–7 months of age, these mice had a tumor incidence of 56%–71% between the two transgenic lines, however tumors were not lethal after 1 year of Env expression and few cells within the tumor expressed high levels of Env. The mouse model we describe here involves ectopic expression of JSRV Env from its cognate LTR-enhancer using an AAV6 vector (A_JE_JJenv). This LTR-enhancer combination has been shown to promote robust transgene expression in the distal airway [15] and likely explains why tumors developed in this immunocompetent mouse model and not in the original model where a weaker promoter was used to drive Env expression. All of the A_JE_JJenv infected C57BL/6 mice developed an antibody-mediated immune response to Env and interestingly, many of the signal transduction pathways analyzed were more strongly activated in immunocompetent versus immunodeficient mice. It is possible that this phenomenon could be due to tumor immunoediting, whereby a more aggressive subpopulation of tumor cells, or one more fit to survive within an immunocompetent host, might have been selected for [34]. However, there was very little difference in the number and size of tumors between C57BL/6 and NOD/SCID mice suggesting that the immune response does not significantly influence the frequency of tumor initiation in this model. Additionally, it is possible that Env-induced tumor cells are expressing immunosuppressive cytokines or chemokines that facilitate escape from immune attack [35].

This virally induced mouse model for lung cancer has several advantages over existing mouse models. For example, this model provides a tractable system that can readily be used in any genetically modified mouse to further dissect the mechanisms involved in lung tumorigenesis without having to generate additional transgenic mice. Unlike known receptor tyrosine kinases and cellular oncogenes, the use of a novel viral oncogene has the potential to uncover new and previously unexplored mechanisms involved in the initiation and progression of lung cancer. Additionally, because JSRV Env is a foreign oncogene it can easily be detected by immunohistochemical staining, allows for live cell sorting of tumor cell populations, and permits investigation into the role of the immune system and chronic inflammation in lung tumorigenesis. Another advantage of our mouse model is that oncogene expression can be “turned on” at any time as defined by the timing of viral vector administration.

In this study, we have identified the PI3K/Akt and MAPK signal transduction pathways as the major pathways involved in JSRV Env-mediated transformation of alveolar type II cells. The Akt pathway is one of the key pathways involved in mediating cell growth, proliferation, survival, metabolism and angiogenesis in human cancers. Akt activation is a frequent and early event in lung tumorigenesis [36], is observed in more than 70% of NSCLC, and is associated with decreased survival [37], [38]. As with our mouse model, Akt activation has been observed in several cell lines transformed by JSRV Env [8]–[11] as well in OPA tumors [39], and is critical for maintaining the transformed phenotype of rodent fibroblast *in vitro*. PDK-1 phosphorylates Akt on Thr308 [40] and mTOR complex 2 (mTORC2) [41] phosphorylates Akt on Ser473. Despite the fact that PDK-1was phosphorylated in C57BL/6 lung tumors and not in the NOD/SCID lung tumors, Akt phosphorylation on residues Ser473 and Thr308 was dramatically increased in both mouse models relative to mock-infected mice suggesting that Akt might be activated independent of PDK-1 in immunodeficient mice. Of note, in contrast to all other reports, an SPC driven JSRV Env expressing transgenic mouse model revealed no significant change in the phosphorylation status of Akt [33].

A marked preference for site-specific phosphorylation of EGFR at Tyr1068 was observed in Env-induced tumors. Autophosphorylation of EGFR on Tyr1068 leads to activation of Akt [25] and since Akt is one of the major mediators of the antiapoptotic effects of EGFR activation [25], it is possible that Env engages this pathway to evade apoptosis.

In addition to promoting cell survival, Akt regulates cell proliferation by phosphorylating a number of targets including the glycogen synthase kinase 3 (GSK3), resulting in GSK3 inhibition [42]. GSK3 promotes cyclin D1 proteolysis; thus by catalyzing GSK3 inhibition through Akt activation, Env may contribute to cyclin D1 accumulation and cell cycle entry [43]. Indeed, cyclin D1 was activated in lung tumors of both C57BL/6 and NOD/SCID mice and cyclin D1 is frequently overexpressed in NSCLC tumours and pre-invasive bronchial lesions [26].

Another downstream target of Akt is mTOR. mTOR regulates translation in response to nutrients and growth factors by phosphorylating components of the protein synthesis machinery, including the protein kinase p70S6K and eukaryotic initiation factor (eIF)4E binding protein-1 (4E-BP1) [44]. mTOR phosphorylation was identified as a downstream target the of the Akt pathway in MDCK and NIH3T3 cells transformed by the JSRV Env protein; however, rapamycin only partially inhibited transformation indicating that another pathway(s) is also involved in JSRV transformation [12], [45]. Similarly, JSRV Env-induced lung tumors expressed higher levels of activated mTOR in both C57BL/6 and NOD/SCID mice relative to mock-infected mice and intense staining was observed in the majority of cells within the tumor. However, the mTOR substrate, p70S6K, which is responsible for 40S ribosomal protein S6 phosphorylation leading to G1 cell-cycle transition [46], was only activated in the NOD/SCID mice suggesting that Env mediated transformation in C57BL/6 mice operates independent of p70S6K, perhaps through inhibition of 4E-BP1.

Negative regulation of the PI3K/Akt pathway is accomplished primarily through the action of the tumor suppressor protein, PTEN. PTEN expression is reduced or lost in a large number of human cancers, including lung cancer [47]. Inactivation of PTEN by phosphorylation results in elevated Akt activity and abnormal growth regulation [48]. Interestingly, PTEN was highly phosphorylated in JSRV Env-induced lung tumors in C57BL/6 mice relative to mock-infected, but the same level of PTEN phosphorylation was not observed in the NOD/SCID mice. Since the total amount of PTEN was nearly equivalent across mice, it would appear that JSRV Env is modulating PTEN post-translationally. In the case of the RJenvC1 lung tumor derived cell line however, PTEN expression levels were significantly reduced relative to the normal lung epithelial cell line suggesting that JSRV Env may be regulating the expression, translation or proteolytic degradation of PTEN.

The second major signal transduction pathway identified in the JSRV Env-induced lung tumors was the MAPK pathway; a family of kinases that play an important role in cell cycle progression, differentiation, transformation, and apoptosis [27]. The MAPK signaling cascade is commonly activated in human lung cancer and members of this pathway, including MEK, have been pursued as potential anticancer targets [49]. Of the three MAPK families that have been characterized, the Erk1/2 and p38 MAPK pathways were found to be activated in JSRV Env-induced lung tumors, particularly in the C57BL/6 mice. Through the use of inhibitors of MEK1 and H/N-Ras, studies have reported the importance of the Erk1/2 signaling in transformation of NIH3T3 and rat kidney epithelial cells (RK3E) cells by the JSRV Env protein [12] and more recently, activation of the Erk pathway has been reported in Env-mediated tumor development in FVB mice [33]. Additionally, OPA tumors stain positive for phosphorylated Erk1/2 [12], [14] indicating that this signaling cascade is of critical importance in JSRV Env-mediated transformation and tumorigenesis.

STAT3 is a latent transcription factor found in the cytoplasm that plays an important role in tumor growth and survival [50]. STAT3 is persistently activated in a number of NSCLC cell lines [51] as well as 50% of lung adenocarcinomas, with a trend toward higher pSTAT3 expression in tumors with bronchioloalveolar carcinoma or adenocarcinoma histology [52]. Phosphorylation of STAT3 at Tyr705 induces dimerization, nuclear translocation and DNA binding while phosphorylation of Ser727 is required for optimal transcriptional activation [53]. STAT3 was phosphorylated on both Tyr705 and Ser727 residues in lungs of A_JE_JJenv infected C57BL/6 mice whereas Tyr705 was not significantly phosphorylated in the A_JE_JJenv infected NOD/SCID mice. In addition to increasing tumor cell proliferation and survival [50], STAT3 also suppresses anti-tumor immunity [54] which might explain why STAT3 was found to be more strongly activated in the C57BL/6 mice. How JSRV Env activates STAT3 is not known, but since the EGFR activated STAT3 pathway [55] is important in NSCLC cell survival [52], Env-induced activation of EGFR could be one mechanism. Constitutive Ser727 phosphorylation of STAT3 in lung tumor cells requires EGFR and MEK kinase activity [56], suggesting that Erk1/2 may also contribute to STAT3 Ser727 phosphorylation in this model. Finally, Src family kinases are known to be involved in STAT3 activation in lung cancer cell lines [28] and since the Src signaling pathway has been implicated in JSRV Env-induced transformation [13], [57], this may represent yet another pathway that could be responsible for STAT3 phosphorylation in JSRV Env-induced lung tumors.

Like STAT3, STAT5 plays an important role in the transduction of oncogenic signals from activated EGFR in NSCLC [58]. Autophosphorylation of EGFR on Tyr1068 leads to an increase in Akt and STAT3/5 activation, both of which are associated with increased NSCLC survival [55]. Therefore, in the case of JSRV Env-induced lung tumors, it is possible that phosphorylation of EGFR on Tyr1068 might mediate an anti-apoptotic effect via STAT3/5 activation.

Elevated tumor COX-2 expression is found in a wide variety of human cancers, including lung cancer [59], and is associated with increased angiogenesis, tumor invasion, suppression of host immunity, resistance to apoptosis [60], and poor survival [61]. In NSCLC cells, COX-2 activates STAT3 by inducing IL-6 expression [62] suggesting that COX-2 upregulation in JSRV Env-induced lung tumors may contribute to STAT3 activation in this model.

The p38 MAPK pathway is often deregulated in cancer; however, the function of p38 MAPK in tumorigenesis is complex. While some cells use this pathway to antagonize cell proliferation and transformation, others subvert this pathway to facilitate proliferation and invasion [63]. Increased levels of phosphorylated p38 MAPK have been correlated with malignancy in lung cancer, indicating that in certain cancers and stage of tumor development, pro-tumorigenic mutations in p38 MAPK pathway can have a selective advantage [64]. In one study, p38 MAPK was shown to be highly activated in life-long nonsmoking patients with lung adenocarcinoma compared to ever smokers [65]. On the other hand, activation of p38 MAPK has been shown to suppress cell growth by inducing apoptosis or senescence in several models of Ras-induced proliferation [66], [67], including lung cancer [68]. In our model, p38 MAPK is highly activated in C57BL/6 lung tumors and Chitra *et al.* also found activated p38 MAPK in the majority of lungs from SPC-JSRV Env transgenic mice [33]. Pharmacological inhibition of p38 MAPK was shown to increase JSRV-Env induced transformation of NIH3T3 and RK3E cells [12] suggesting that p38 MAPK may be acting as a tumor suppressor in JSRV-Env induced lung tumors.

**Figure 6:**
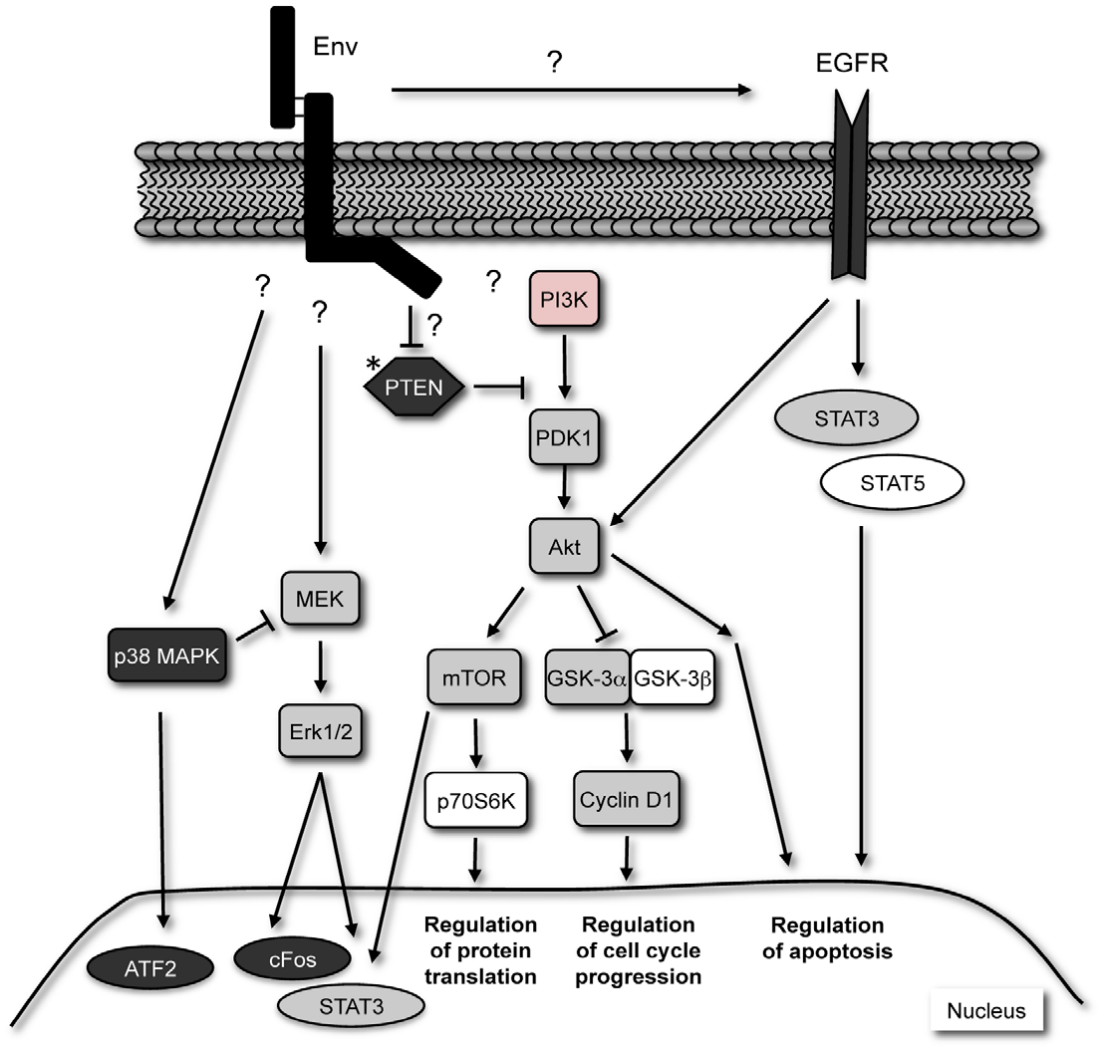
Signaling pathways activated in JSRV Env-induced lung tumors. The JSRV Env protein leads to activation of signals through both the PI3K/Akt and MAPK pathways leading to cell cycle progression, regulation of protein translation and evasion of apoptosis. Kinases are represented by squares, phosphatases by diamonds, and transcription factors by circles. Possible interactions, or other unidentified factors presumed to be involved in the transformation by JSRV Env are indicated with questions marks (?) or by a red colored box, as in the case of PI3K. Signaling molecules activated exclusively in the NOD/SCID mouse model are depicted in white. Signaling molecules uniquely activated in the C57BL/6 mouse model and RJenvC1 tumor cell line are depicted in black. Signaling molecules activated in all three models are depicted in grey. An asterisk on PTEN indicates that gene expression reduced in the RJenvC1 tumor cell line. Note that in the absence of functional assays, such as those involving inhibitors, the arrows and inhibition bars are only speculative for the tumors reported here.

The transcription factor ATF2 is responsive to a host of environmental and cellular stimuli ranging from mitogenic signals via regulation through Erk or stress signals via regulation through p38 MAPK [69]. The fact that phospho-ATF2 is more highly activated in the C57BL/6 mice suggests that the stress-activated p38 MAPK pathway could be responsible for its activation.

An overview of the signaling pathways identified in this study to be involved in JSRV Env-induced lung cancer is shown in Figure 6. Of note, a number of key pathways activated in lung cancer in never smokers [16], including EGFR, Akt, MAPK and STAT3, are activated in this mouse model. In view of the similarities to human lung adenocarcinoma this model may serve to increase our understanding of the molecular pathogenesis of human lung adenocarcinoma, particularly that of never smokers. Moreover, understanding the mechanisms involved in JSRV-Env induced tumorigenesis may lead to the discovery of novel pathways active in lung cancer and could lead to improved diagnosis and treatment of lung cancer in humans. Lastly, as viruses are increasingly being associated with human cancer [70], including JSRV and human lung cancer [5], [71], [72], it is important to understand the underlying mechanisms by which viruses cause cancer.
